# Role of the Wnt/β-Catenin Signaling Pathway in Mediating Outer Root Sheath Stem Cells to Promote Hair Follicle Regeneration and Skin Wound Healing in Mice

**DOI:** 10.3390/cells15111038

**Published:** 2026-06-05

**Authors:** Hangzhen Zhou, Jiaxin Liu, Lie Yang, Shan Li, Shuwei Li

**Affiliations:** 1 Key Laboratory of Protection and Utilization of Biological Resources in Tarim Basin of Xinjiang Production and Construction Corps, College of Life Science and Technology, Tarim University, 705 Hongqiao South Road, Alar 843300, China; 120100051@taru.edu.cn (H.Z.); 120120003@taru.edu.cn (J.L.); 120220041@taru.edu.cn (L.Y.); 2 State Key Laboratory for Development and Utilization of Forest Food Resources, College of Food and Health, Zhejiang Agriculture and Forestry University, 666 Wusu Road, Hangzhou 311300, China

**Keywords:** hair follicle stem cells, Wnt/β-catenin signaling pathway, outer root sheath, transplant, wound healing

## Abstract

**Highlights:**

**What are the main findings?**

- We analyzed the refined structural changes during hair follicle development and compared the protein expression of hair follicle stem cells in vitro and in vivo. Hair follicle stem cells promote skin and hair follicle growth through proliferation, differentiation, and migration.
- When hair follicles are damaged or degenerated, activating the Wnt/β-catenin sig -naling pathway promotes the activation and proliferation of hair follicle stem cells.
- The outer root sheath houses the stem cell niche at the hair follicle bulge. In this study, the outer root sheath was successfully transplanted subcutaneously into mice, where it regenerated into complete hair follicles. Hair follicle stem cells demonstrated enhanced regenerative and tissue-repair capabilities within damaged outer root sheaths.

**What are the implications of the main findings?**

- Damage to the skin or hair follicles activates these stem cells, which then help repair the injured tissue.
- Activating the Wnt/β-catenin signaling pathway facilitates the growth and regeneration of both the skin and hair follicles, thereby aiding tissue repair.
- Transplantation of the outer root sheath can effectively promote tissue repair.

**Abstract:**

Hair follicle (HF) stem cells are multipotent adult stem cells that play a key role in the hair follicle cycle. However, it remains poorly understood how the outer root sheath (ORS)—specifically, the stem cells in the bulge region of the hair follicle—promotes skin repair. This study aims to investigate the role of bulge stem cells in tissue growth and repair and to determine whether the ORS of transplanted hair follicles can facilitate skin repair. We further seek to elucidate the mechanisms by which bulge stem cells contribute to hair follicle development, regeneration, and skin wound healing. In this study, hair follicle samples were obtained from neonatal mice using microdissection. Hair follicle morphology was assessed by Sirius red staining, H&E staining, and transmission electron microscopy. Immunofluorescence staining was used to detect changes in CD34 and SOX9 protein expression. Additionally, microdissection-based hair follicle transplantation and Western blotting were employed to investigate protein activation and inhibition in the Wnt/β-catenin signaling pathway. The results show that the hair follicle bulge, inner root sheath, and dermal papilla all increase in size as hair follicles grow, with each structure growing relatively rapidly on day 7. Treatment with Teplinovivint effectively inhibits the expression of Wnt/β-catenin signaling pathway-related proteins and hair follicle stem cell markers. Damaged hair follicle tissues cultured in vitro are capable of self-repair. At the transplantation site, the skin gradually closes as the outer root sheath wound heals. In contrast, the central region of the outer root sheath becomes progressively filled with numerous dividing cells and extracellular matrix. The inner portion of the outer root sheath is densely populated with cells, and the markers CD34 and SOX9 are also widely distributed. This indicates that activation of the Wnt/β-catenin signaling pathway enhances the proliferation and differentiation of hair follicle stem cells, thereby promoting hair follicle growth, repair of damaged follicles, and healing of skin wounds. Furthermore, this study demonstrates the feasibility of using transplanted outer root sheath (ORS) to repair skin wounds—specifically, the potential to achieve large-scale hair regeneration from a limited number of hair follicle stem cells—providing a new approach for the clinical treatment of skin injury disorders. Nevertheless, achieving long-term, stable, and scalable clinical translation of ORS stem cells for hair follicle regeneration remains a major challenge.

## 1. Introduction

The hair follicle is a mini-organ composed of various cells, resulting from epithelial–mesenchymal interactions [1]. As a stem cell niche, the hair follicle harbors numerous undifferentiated stem cells, among which hair follicle stem cells and dermal papilla cells are the most important. Hair follicle stem cells proliferate during anagen of the hair cycle, migrate to the bulb region in catagen to form new matrix cells, and remain in a slow-cycling or quiescent state during telogen. The proliferation and differentiation of hair follicle stem cells help maintain hair follicle homeostasis [2] and promote skin wound healing [3]. In addition, hair follicle stem cells can secrete cytokines to modulate the niche of surrounding cells and tissues [4,5,6]. Numerous markers can be used to identify hair follicle stem cells in the bulge region, such as CK15, CK19, CD34, CD200, Lgr5, and SOX9 [7,8,9].

Wnt signaling is divided into two pathways: the canonical (or Wnt/β-catenin -dependent) pathway and the non-canonical pathway. The canonical Wnt signaling pathway primarily regulates cell proliferation, whereas the non-canonical pathway regulates cell polarity and motility. The Wnt/β-catenin pathway consists of four components: extracellular signaling molecules, membrane receptor binding, intracellular signal transduction, and nuclear gene transcription. Wnt family proteins primarily mediate extracellular signaling. The dominant receptors in the membrane receptor binding step include the co-receptor protein FZD and the co-receptors LRP5 and LRP6. The intracellular signal transduction component comprises proteins such as β-catenin, DVL, GSK-3β, AXIN, APC, and CKI. The nuclear transcriptional part consists of β-catenin protein translocated to the nucleus, members of the TCF/LEF family, and downstream target genes such as MMPs and c-Myc. MMPs and c-Myc are involved in regulating cell proliferation, migration, and invasion. The translocation of β-catenin protein from the cytoplasm to the nucleus is the main feature of activation of the Wnt/β-catenin signaling pathway.

The Wnt/β-catenin signaling pathway is highly conserved across complex organisms and plays a key role in regulating fundamental physiological processes, including cell proliferation, differentiation, migration, and programmed cell death [10,11,12,13]. In hair follicles, the canonical Wnt signaling pathway plays an important role in regulating the proliferation and fate determination of hair follicle stem cells. Wnt3a and β-catenin in hair matrix cells accelerate the hair follicle cycle and promote hair regeneration by activating the Wnt/β-catenin signaling pathway [14,15,16,17]. During skin wound healing, activation of the Wnt pathway in the dermis inhibits adipogenic differentiation of dermal fibroblasts. It induces lipolysis and dedifferentiation of mature adipocytes, both processes that facilitate myofibroblast formation [18].

The Wnt/β-catenin signaling pathway regulates cellular life activities and processes and plays a crucial role in tissue and organ development and regeneration. The mechanisms by which hair follicle stem cells repair skin and hair follicles are not yet fully understood, and it is necessary to clarify the role of the Wnt/β-catenin signaling pathway in the wound healing mediated by these stem cells. Therefore, this study investigates how the Wnt/β-catenin signaling pathway promotes tissue repair through hair follicle stem cells. Through animal and cellular experiments, we elucidate the mechanism by which the Wnt/β-catenin signaling pathway enhances the therapeutic potential of hair follicle stem cells in tissue injury. This research provides a theoretical basis for using hair-follicle ORS to repair skin wounds and offers novel therapeutic tools for the clinical treatment of skin diseases. Additionally, the role of the Wnt/β-catenin signaling pathway in promoting skin healing opens new directions for the development of targeted drugs.

## 2. Materials and Methods

### 2.1. Sample Collection and Grouping

#### 2.1.1. Specimen Preparation and Sampling

A total of 18 suckling mice at 3, 7, and 15 days postnatal were randomly selected, with 6 mice at each time point. The mice were anesthetized via intraperitoneal injection of ketamine (200 mg/kg) and then euthanized by rapid cervical dislocation. Under a microscope, the vibrissae hair follicles were carefully dissected using surgical scissors, ophthalmologic forceps, and syringe needles. The obtained hair follicle samples were placed in a universal tissue fixative solution and stored at 4 °C for further use.

Since the hair follicles of adult mouse vibrissae have fully developed and the hair follicle stem cells remain quiescent most of the time, it is difficult to investigate the effect of hair follicle stem cells on vibrissae hair follicle growth accurately. Therefore, vibrissae hair follicles from young mice at the growing stage were selected for the experiment. Adult C57BL/6J mice were purchased from the Animal Experiment Center of Xinjiang Medical University. All mice were fed SPF-grade clean feed in the Key Laboratory of the Xinjiang Production and Construction Corps at Tarim University. The temperature was maintained at 22–26 °C, and the relative humidity at 30–50%. During the experiment, adult mice had free access to food and water, and suckling mice had free access to food. The experiment randomly selected growing suckling mice weighing 3.28 ± 0.4 g. The neonatal mice used in the experiment were not sexed. This animal experiment was approved by the Ethics Committee of Tarim University (Ethics Number: DWLL202312011).

#### 2.1.2. Taken Samples for Western Blot

Collection of hair follicles from autologous neonatal mice aged 3, 7, and 15 days; mice were randomly selected and divided into a control group and an inhibitor group, with 6 mice per group at each time point. The control group received injections of PBS buffer (25 μL per side) into the mouse vibrissae follicles using insulin needles. The inhibitor group received injections of the Teplinovivint inhibitor solution (25 μL per side) into the vibrissae using insulin needles. The Teplinovivint inhibitor stock solution was prepared at a concentration of 1 mg/mL in PBS (pH 7.4). The compound (HY-137454, purity 98.01%) was purchased from MedChemExpress (Monmouth Junction, NJ, USA). Sampling was performed 24 h after injection. After euthanizing the mice, the vibrissae follicles were dissected. The number of hair follicle samples obtained at each time point was consistent across groups. The samples were then placed in cryotubes and stored in liquid nitrogen for future use.

#### 2.1.3. Collection of Hair Follicles Cultured in Vitro

Due to the small size and morphological challenges of vibrissae hair follicles in neonatal mice within 2 days after birth, dissection and isolation were difficult. In contrast, the vibrissae hair follicles of 3-day-old newborn mice exhibited clear outlines, facilitating isolation and extraction, while hair follicle stem cells maintained high viability. Therefore, 3-day-old newborn mice were randomly selected and euthanized. Under sterile conditions, the vibrissae hair follicles were dissected after disinfection. An equal number of hair follicles were randomly selected based on the experimental duration and cultured for 1, 3, and 5 days in a 37 °C, 5% CO_2_ incubator. The follicles were divided into a control group and an inhibitor group, with consistent numbers of hair follicles at each time point and three replicates per group. The control group was cultured in 35 mm dishes containing 2 mL of complete medium, while the inhibitor group was cultured in 2 mL of inhibitor medium. The medium was replaced daily. The cultured hair follicle samples obtained at each time point were placed in cryotubes and stored in liquid nitrogen for future use.

#### 2.1.4. The Outer Root Sheath Transplantation Experiment

Microdissection techniques were used to obtain randomly selected 7-day-old C57BL6J neonatal mouse hair follicles (donor mice). The hair papilla was removed, and the hair shaft and inner root sheath were pulled out to obtain the outer root sheath of the hair follicles. Then, it was transplanted into the dermis of the back skin of randomly selected 6-week-old C57BL6J neonatal mice (recipient mice). Inhibitor treatment group: Once a day, 25 μL of inhibitor working solution was injected into the skin wound of mice with transplanted outer root sheaths. EdU treatment group: 25 μL of EdU working solution was injected into the skin wounds of mice with transplanted outer root sheaths, and samples were taken 24 h later.

### 2.2. Preparation of Hair Follicle Sections

The reserved vibrissae hair follicle samples from Section 2.1 were used as materials for preparing frozen sections. The cryostat was pre-cooled, and the section thickness was set to 10 μm. The machine was ready for use when the temperature of the sectioning chamber dropped below −20 °C. The OCT embedding compound was poured into the embedding molds. Individual hair follicle samples were placed vertically or horizontally into the embedding compound, parallel or perpendicular to the mold. The entire mold was clamped into liquid nitrogen for rapid freezing. Once the embedding compound turned white and hardened, the mold was transferred to the cryostat. The mold was pressed to detach the embedding compound, which was then fixed onto the specimen stage. After embedding the specimen stage into the cryostat, sections were cut by manually rotating the wheel. The resulting sections were placed in section boxes and stored at −20 °C for subsequent experiments.

In each group of 6 mice, at least 3 hair follicles/per mouse were selected and embedded in OCT. Three similar frozen sections could be made from the embedded hair follicles in each mold. A total of 48 hair follicles in each group of mice were ultimately analyzed. The images of the sections were collected using a laser confocal microscope without blind analysis. The same microscope parameters were set, and Image J 1.54 ’s default threshold was used. The intra-group correlation coefficient evaluated the consistency of the images.

### 2.3. Sirius Red Staining of Hair Follicles

The frozen sections preserved in Section 2.2 were taken out from the −20 °C freezer and allowed to reach room temperature. They were fixed with tissue fixative for 15 min and then rinsed under running water. The sections were stained in Sirius red staining solution for 8 min, followed by sequential rinsing and dehydration in three dehydration tanks containing 500 mL of absolute ethanol for 5 s each. The sections were then placed in clean xylene for 5 min for transparency and mounted with neutral balsam. Microscopic examination and image acquisition analysis were performed.

### 2.4. Hematoxylin-Eosin (H&E) Staining and Transmission Electron Microscopy of Hair Follicles

#### 2.4.1. H&E Staining

The frozen sections preserved in Section 2.2 were taken out from the −20 °C freezer and allowed to reach room temperature. They were fixed with tissue fixative for 15 min and rinsed under running water. The sections were treated with a high-definition constant staining pretreatment solution for 1 min. Subsequently, the sections were sequentially immersed in hematoxylin staining solution for 5 min, rinsed with tap water for 3 min, differentiated in differentiation solution for 3 s, rinsed with tap water for 5 min, treated with blue returning solution for 5 min, and rinsed under running water for 3 min. The sections were then dehydrated in 95% ethanol for 1 min and stained with eosin for 15 s. Three dehydration tanks containing 500 mL of absolute ethanol were labeled I, II, and III. The sections were sequentially immersed in absolute ethanol I for 2 min, then in absolute ethanol II for 2 min, and finally in absolute ethanol III for 2 min. After completion, the sections were mounted with neutral balsam. Microscopic examination and image acquisition analysis were performed.

#### 2.4.2. Transmission Electron Microscopy Imaging

Fresh hair follicles were obtained using the method described in Section 2.1, and the isthmus tissue of the fresh hair follicles was carefully excised. Mechanical damage, such as stretching, contusion, and compression, was minimized during the process. Sampling was completed within 3 min, and the tissue size was approximately 1 mm^3^. Before sampling, a culture dish containing electron microscopy fixative was prepared. Immediately after excision, the small tissue block was placed into the culture dish. Using a surgical blade, the tissue was further trimmed into 1 mm^3^; blocks within the fixative in the culture dish. The trimmed tissue blocks were then transferred to microcentrifuge tubes containing fresh electron microscopy fixative for continued fixation, and the tubes were stored at 4 °C. Before use, the tissue blocks were rinsed three times in PBS buffer for 15 min each. They were then fixed in 1% osmium tetroxide (prepared in PBS buffer) for 2 h at room temperature in the dark, followed by three 15 min rinses in PBS buffer. The tissues were sequentially dehydrated in an ascending series of ethanol concentrations (30%, 50%, 70%, 80%, 95%, 100%, and 100%) for 20 min each step, followed by two treatments in 100% acetone for 15 min each.

The tissues were first immersed in a mixture of acetone and 812 embedding agent (1:1 ratio) and placed in a 37 °C oven for 3 h. They were then transferred to a mixture of acetone and 812 embedding agent (1:2 ratio) and incubated overnight at 37 °C in an oven for infiltration. Finally, pure 812 embedding agent was poured into embedding molds, and the samples were inserted into the molds and placed in a 37 °C oven overnight. The embedding molds were placed in a 60 °C oven for 48 h to polymerize, and the resulting resin blocks were reserved for later use. The resin blocks were sectioned into 80 nm ultrathin sections using an ultramicrotome. The sections were collected on 150-mesh Formvar-coated copper grids. The grids were stained with 2% uranyl acetate in saturated alcohol solution for 8 min in the dark, rinsed three times with 70% ethanol, and three times with ultrapure water. They were then stained with 2.6% lead citrate solution for 8 min while avoiding carbon dioxide exposure, rinsed three times with ultrapure water, and lightly dried with filter paper. The grid sections were placed in grid boxes and allowed to dry overnight at room temperature. The samples were observed under a transmission electron microscope at the analysis and testing center, and images were acquired for analysis.

### 2.5. EdU Labeling Staining of Hair Follicles

Newborn mice aged 3, 7, and 15 days were randomly selected as described in Section 2.1. Each mouse was injected with 25 μL of EdU labeling solution into both left and right vibrissae using insulin needles. Sampling was performed 24 h post-injection, with 6 mice per time point. Frozen sections were prepared as described in Section 2.2 for EdU labeling and staining. The frozen sections were thawed to room temperature and fixed with an appropriate amount of fixative for 15 min. The fixative was removed, and the sections were washed three times with PBS buffer for 5 min each. After removing the PBS buffer, a permeabilization solution was applied to cover the tissue, and the tissue was incubated for 15 min at room temperature. The permeabilization solution was then removed, and the sections were washed twice with PBS buffer for 5 min each. During fixation and permeabilization, the click reaction solution was prepared according to the manufacturer’s instructions (freshly prepared before use). After removing the PBS buffer, the click reaction solution was added to the sections and gently shaken to ensure complete coverage of the cells or tissue. The sections were incubated for 30 min at room temperature in the dark. The click reaction solution was removed, and the sections were washed three times with PBS buffer for 5 min each. The PBS buffer was then removed, and Hoechst 33342 (Merck KGaA, Darmstadt, Germany) staining solution, diluted 1:500 in PBS buffer, was added to cover the cells, followed by a 5 min incubation. The Hoechst 33342 staining solution was removed, and the sections were washed three times with PBS buffer for 5 min each. After briefly air-drying the sections, an anti-fade mounting medium and neutral balsam were applied. The mounted samples were examined using a laser scanning confocal microscope at the analysis and testing center. The percentage of proliferating cells was analyzed using Image J 1.54 software.

### 2.6. CD34 and SOX9 Immunofluorescence Staining of Hair Follicles

The frozen sections preserved in Section 2.2 were placed in a 37 °C oven for 20 min and dried. After removing moisture, the sections were fixed with fixative for 30 min and then washed three times with PBS buffer on a decolorizing shaker for 5 min each. The PBS buffer was removed, a permeabilization solution was applied to cover the tissue, and the tissue was incubated for 15 min at room temperature. The permeabilization solution was removed, and the sections were washed three times with PBS buffer for 5 min each. After a brief air-drying, a hydrophobic barrier was drawn around the tissue sections using a hydrophobic barrier pen. Then, 3% BSA was added to block the sections for 30 min. Primary antibodies were prepared as follows: CD34: antibody diluent = 1:200; SOX9: antibody diluent = 1:200. After briefly air-drying the sections, the prepared primary antibodies were added dropwise. The sections were placed flat in a humidified chamber and incubated overnight at 4 °C. The slides were washed three times with PBS buffer on a decolorizing shaker for 5 min each. Secondary antibodies were prepared as follows: Goat Anti-Rabbit IgG H&L: antibody diluent = 1:500. After briefly air-drying the sections, the corresponding secondary antibodies were added and incubated for 50 min at room temperature in the dark. The slides were washed three times with PBS buffer for 5 min each. After briefly air-drying the sections, DAPI staining solution was added, and the sections were incubated for 10 min at room temperature in the dark. The slides were washed three times with PBS buffer on a decolorizing shaker for 5 min each. After briefly air-drying the sections, an anti-fade mounting medium and neutral balsam were applied. The mounted samples were examined using a laser scanning confocal microscope at the analysis and testing center. The average fluorescence intensity of the samples was calculated using Image J 1.54 software, and expression changes were analyzed.

### 2.7. Protein Extraction from Hair Follicles and Western Blot Analysis

Protein was extracted from the hair follicle samples preserved in Section 2.1. The samples were placed in 1.5 mL RNase-free centrifuge tubes, to which RIPA tissue lysis buffer, PMSF protease inhibitor, and three 3 mm grinding beads were added. The mixture was homogenized using a frozen tissue homogenizer and then lysed on ice for 30 min. After homogenization, the samples were centrifuged at 12,000 rpm for 10 min in a refrigerated centrifuge, and the supernatant was transferred to a new 1.5 mL centrifuge tube. The total protein concentration of each sample was measured using a BCA protein concentration assay kit, and the concentrations were standardized. A 5× reducing protein loading buffer was added at a 4:1 ratio, and the mixture was denatured at 100 °C for 10 min before being stored at −80 °C. An SDS-PAGE color gel ultra-fast preparation kit was used to prepare the protein electrophoresis gel, consisting of a 12% separation gel and a 5% stacking gel. The protein samples were loaded into the gel wells, and electrophoresis was performed at 80 V for the stacking gel and 120 V for the separation gel. A PVDF membrane was selected for transfer, and a standard wet transfer apparatus was used to transfer the membrane at 120 V for 60 min in an ice bath. After transfer, the protein membrane was immediately placed in Western wash buffer and rinsed for 2 min to prevent drying. The wash buffer was removed, and the Western blocking buffer was added. The membrane was gently agitated and blocked at room temperature for 60 min. The blocking buffer was then removed, and primary antibodies against Wnt3a, Wnt5a, β-catenin, CD34, CD200, Nanog, OCT4, SOX2, SOX9, and GAPDH (1:1000 dilution) were added separately. The membrane was incubated overnight at 4 °C. After incubation, the membrane was washed 3 times with sterile PBS, then incubated with HRP-conjugated secondary antibody in dilution buffer at room temperature for 2 h. Finally, the membrane was washed three times with sterile PBS. GAPDH was used as the internal reference protein. Images were captured using a WB imaging system, saved, and analyzed in Image J 1.54 to obtain grayscale values.

### 2.8. Statistical Analysis

All data in this study are expressed as mean ± SEM. Data were analyzed using GraphPad Prism 9.0. For each experiment, biologically independent replicates (*n* = 6) and technical replicates (*n* = 3) were performed. Differences between two groups were analyzed using an unpaired two-tailed *t*-test. A *p*-value < 0.05 was considered statistically significant. Repeated-measures ANOVA was applied to continuous variables collected at multiple time points from multiple groups, assuming equal sample sizes and homogeneity of variance. Tukey’s method was then used for post hoc comparisons.

## 3. Results

### 3.1. Comparison of Fine Structural Changes and Hair Follicle Stem Cell Expression During Hair Follicle Development

**Figure 1:**
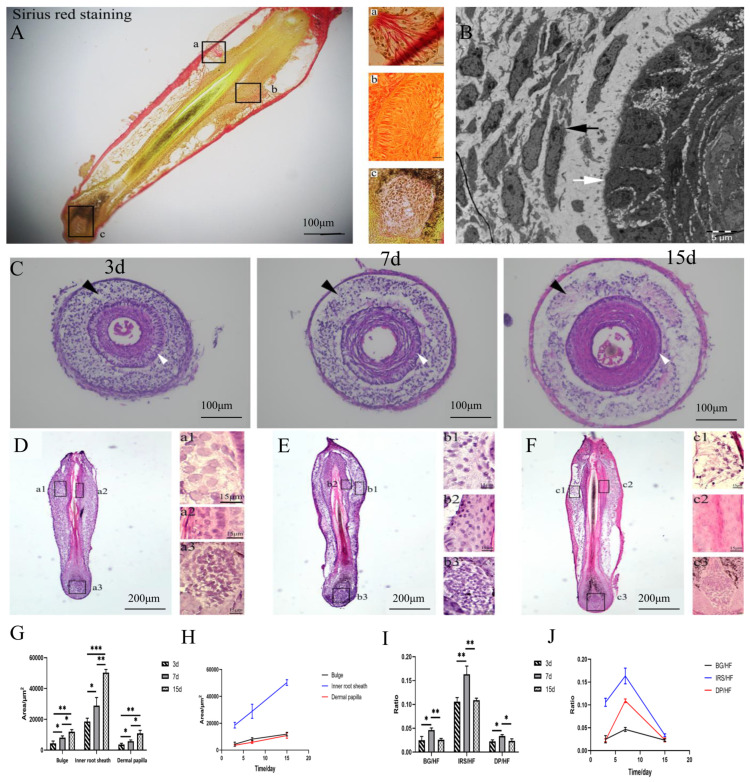
(**A**) Sirius red staining of mouse vibrissa hair follicles. Red indicates collagen fibers. Scale bars: 100 μm (overview); (**a**) Hair follicle bulge, 5 μm; (**b**) Inner root sheath, 10 μm; (**c**) Dermal papilla, 15 μm. (**B**) Transmission electron micrograph of the inner and outer root sheaths of the hair follicle. Black arrow: Outer root sheath cells; White arrow: Henle’s layer cells of the inner root sheath. Scale: 5 μm. (**C**) Cross-sectional morphology of hair follicles at different growth stages. Black arrow: Outer root sheath; White arrow: Inner root sheath (from outer to inner: Henle’s layer, Huxley’s layer, and sheath cuticle). Scale: 100 μm; (**D**–**F**) HE staining of hair follicles at different growth stages and corresponding higher-magnification views. (**D**) Hair follicle at day 3; (**E**) day 7; (**F**) day 15. (**a1**–**c1**) Hair follicle bulge; (**a2**–**c2**) Inner root sheath; (**a3**–**c3**) Dermal papilla. Scale bars: 200 μm (whole follicles), 15 μm (insets); (**G**–**J**) Changes in the area and proportional area of hair follicle structures. (**G**) Statistical analysis of the area of hair follicle structures at different time points; (**H**) Changes in the area of hair follicle structures; (**I**) Statistical analysis of the proportional area of hair follicle structures at different time points; (**J**) Changes in the proportional area of hair follicle structures. *: *p* < 0.05; **: *p* < 0.01; ***: *p* < 0.001. Each experiment included 6 biological replicates and 3 technical replicates.

The fine structure of the hair follicle is shown in Figure 1A. Collagen fibers, stained red, are distributed along the outer side of the hair follicle, enclosing the entire structure. The outer root sheath appears loosely organized, with the bulge region fanning out along the isthmus of the hair follicle. In contrast, the inner root sheath is tightly arranged and surrounds the hair shaft, which contains melanin. The hair bulb exhibits a hair matrix rich in melanocytes that envelops the dermal papilla. As hair follicles develop, the areas of the bulge, inner root sheath, and dermal papilla significantly increase with the growth of newborn mice (*p* < 0.05). Among these, the inner root sheath shows the highest rate of expansion, while the bulge and dermal papilla exhibit slower growth rates (Figure 1B,C). Over time, cell distribution within the outer root sheath becomes progressively sparser. The cells in this region are polygonal with large intercellular spaces, creating numerous gaps that enhance the flexibility of the outer root sheath. Conversely, cells in the inner root sheath are densely packed. The outermost Henle’s layer consists of flattened, interconnected cells, which encircle the tile-like Huxley’s layer, collectively maintaining the structural integrity of the hair follicle. As hair follicles grow, the proportional areas of these three regions initially increase and then decrease, with significant differences observed across time points (*p* < 0.05). On day 7, the proportional areas of all three regions are higher than those on day 3 and day 15. In the early stages of hair follicle development, growth is primarily driven by the expansion of the bulge, inner root sheath, and dermal papilla. Subsequently, the growth rates of other tissues within the hair follicle gradually increase, promoting overall hair follicle development. Statistical data are summarized in Table 1, and experimental results are illustrated in Figure 1D–J.

**Figure 2:**
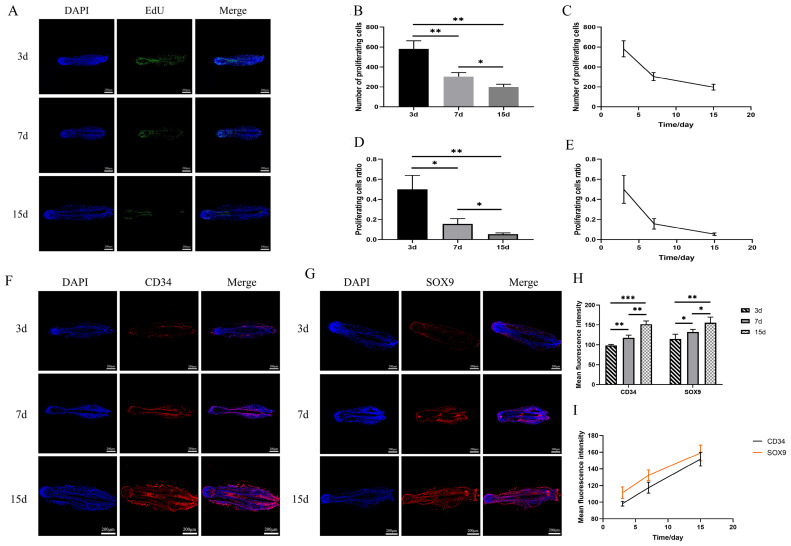
(**A**) Detection of proliferating cells in the hair follicle at different time points. (Green: EdU; Blue: DAPI-labeled nuclei), Scale bar: 200 μm. (**B**–**E**) Changes in the number and proportion of proliferating cells in hair follicle at different time points. ((**B**,**C**) Statistical analysis and changes in the number of proliferating cells; (**D**,**E**) Statistical analysis and changes in the proportion of proliferating cells. *: *p* < 0.05; **: *p* < 0.01. (**F**,**G**) Immunofluorescence staining of CD34 and SOX9 in hair follicle at different time points, (Red: CD34, SOX9; Blue: nucleus (DAPI); scale: 200 μm). (**H**,**I**) Statistical analysis and changes in the mean fluorescence intensity of CD34 and SOX9 in hair follicles at different time points, *: *p* < 0.05; **: *p* < 0.01; ***: *p* < 0.001.

**Figure 3:**
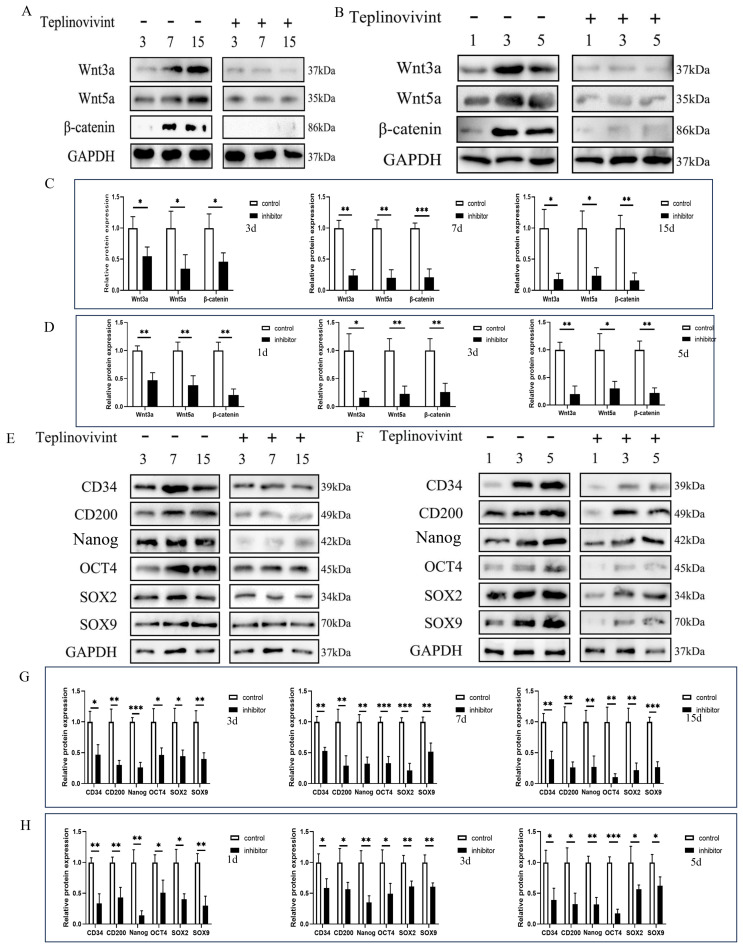
Comparison of the expression of Wnt signaling pathway-related proteins between the control group and the inhibitor group during the growth process of autologous hair follicles and hair follicles in vitro. −: no inhibitor added; +: add inhibitors. (**A**) Banding pattern of Wnt signaling pathway related proteins in the control group and the inhibitor group in the autologous hair follicles. (**B**) Banding pattern of Wnt signaling pathway related proteins in the control group and the inhibitor group in the hair follicle in vitro. (**C**) Protein grayscale statistics of autologous hair follicle growth about (**A**). (**D**) Protein grayscale statistics of hair follicle in vitro about (**B**). (**E**) Comparison of the expression of stem cell related proteins between the control group and the inhibitor group during the growth process of autologous hair follicles. (**F**) Comparison of the expression of stem cell related proteins between the control group and the inhibitor group during hair follicle culture process in vitro. (**G**) Protein grayscale statistics of hair follicle growth about (**E**). (**H**) Protein grayscale analysis of hair follicle and culture about (**F**). *: *p* < 0.05; **: *p* < 0.01; ***: *p* < 0.001.

At 3 days, proliferating cells were widely distributed within the hair follicles. By 7 days, these cells were predominantly observed in the lower segment of the hair follicles, whereas at 15 days their numbers decreased and they were mainly concentrated in the hair bulb region. This suggests that the hair bulb primarily regulates the later stages of hair follicle development. Over time, both the number and proliferation rate of hair follicle cells significantly decreased (*p* < 0.05), indicating a decline in hair follicle cell proliferative capacity during development and a reduction in newly generated cells. However, for the number of proliferating cells, there was an extremely significant difference between day 3 and days 7 and 15 (*p* < 0.01), and a significant difference between days 7 and 15 (*p* < 0.05). For the proliferating cell ratio, a highly significant difference was observed between day 3 and day 15 (*p* < 0.01). In contrast, significant differences were observed between day 3 and day 7 and between day 7 and day 15 (*p* < 0.05). The results are shown in Figure 2A–E. The positive signals for CD34 and SOX9 intensified over time, with a gradual increase in the area of positive staining. The average fluorescence intensity of CD34 and SOX9 markers also increased significantly with time (*p* < 0.05), as shown in Figure 2F–I. During hair follicle growth, the expression levels of CD34 and SOX9 proteins gradually increased, reflecting enhanced activity of hair follicle stem cells. This indicates that hair follicle stem cells play a promoting role in hair follicle development and growth, both in vivo and in vitro. Teplinovivint inhibited the Wnt/β-catenin signaling pathway in hair follicles, showing a significant difference compared to the control group (*p* < 0.05), as illustrated in Figure 3A–D. Compared to the control group, Teplinovivint significantly reduced the expression of hair follicle stem cell marker proteins in both in vivo and in vitro cultured hair follicles (*p* < 0.05), as shown in Figure 3E–H.

### 3.2. Activation of the Wnt/β-Catenin Signaling Pathway Mediates Hair Follicle Stem Cells in Promoting Repair of Injured Hair Follicles

**Figure 4:**
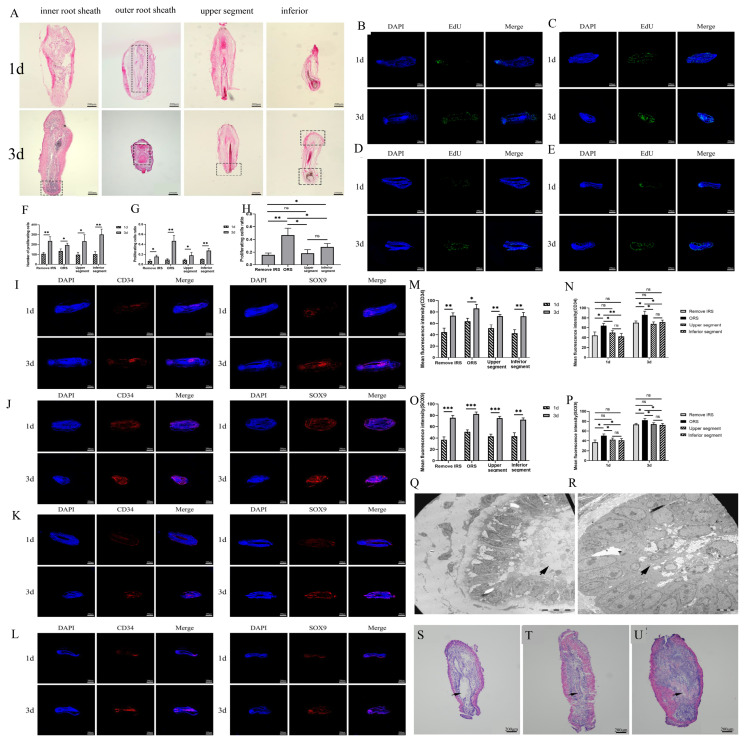
(**A**) Morphological changes in different damaged tissues of hair follicle cultured in vitro, (Dashed box: major changing positions in each tissue; Scale: 200 μm). (**B**–**E**) Detection of proliferating cells in different damaged tissues of hair follicle cultured in vitro. ((**B**) Remove the hair follicle of the inner root sheath; (**C**) Outer root sheath of the hair follicle; (**D**) The upper segment of the hair follicle; (**E**) The inferior segment of the hair follicle; Green: EdU; Blue: Nucleus (DAPI); Scale: 200 μm). (**F**–**H**) Changes in the number and proportion of proliferating cells in different damaged tissues of hair follicle cultured in vitro, (Remove IRS: Remove the hair follicle of the inner root sheath; ORS: Outer root sheath of the hair follicle; Upper segment: The upper segment of the hair follicle; Inferior segment: The inferior segment of the hair follicle. (**F**) Statistics and changes in the number of proliferating cells; (**G**) Statistics and changes in the proportion of proliferating cells; (**H**) Comparison of the proportion of proliferating cells in different damaged tissues on the 3 days of culture. *ns*: *p* > 0.05; *: *p* < 0.05; **: *p* < 0.01. (**I**–**L**) Immunofluorescence staining of CD34 and SOX9 in the hair follicle. (**I**) Inner root sheath; (**J**) Outer root sheath; (**K**) Upper segment of the hair follicle; (**L**) Inferior segment of the hair follicle. Red: CD34 and SOX9; Blue: Nuclei (DAPI); Scale bar: 200 μm. (**M**–**P**) Changes in mean fluorescence intensity of CD34 and SOX9 in different injured regions of hair follicles cultured in vitro. IRS, Inner root sheath; ORS, Outer root sheath; Upper segment; Inferior segment. (**M**) Statistical analysis of mean fluorescence intensity (MFI) for CD34; (**N**) Comparison of CD34 MFI among different damaged tissues; (**O**) Statistical analysis of MFI for SOX9; (**P**) Comparison of SOX9 MFI among different damaged tissues. *ns*: *p* > 0.05; *: *p* < 0.05; **: *p* < 0.01; ***: *p* < 0.001. (**Q**,**R**) Transmission electron microscopy images of the hair follicle outer root sheath before and after in vitro culture. (**Q**) Outer root sheath before culture, Scale bar 10 μm. (**R**) Outer root sheath after culture, scale bar 5 μm. The black arrow indicates the center of the outer root sheath. (**S**–**U**) Morphological changes in the outer root sheath of hair follicles cultured in vitro from the Wnt/β-catenin signaling pathway inhibition group. (**S**) Cultured for 0 days; (**T**) Cultured for 1 day; (**U**) Cultured for 3 days. Black arrows indicate the main sites of change; Scale bar: 200 μm. Each experiment included 6 biological replicates and 3 technical replicates.

With prolonged culture time, the number of cells increased at the dermal papilla region in the inferior segment of the inner root sheath-removed hair follicles, and the dermal papilla gradually formed. The wound in the outer root sheath of the hair follicle closed, and the central structure changed, exhibiting two cell masses formed by the aggregation of numerous cells, which were surrounded by a layer of tightly arranged cells. The incision in the upper segment of the hair follicle gradually closed, and the composition and morphology of the hair shaft at the incision site changed. The incision in the inferior segment of the hair follicle gradually closed, forming an outer root sheath that enveloped the entire tissue. Meanwhile, connective tissue in the inferior segment increased, and melanin accumulated in the hair bulb, surrounding the dermal papilla (Figure 4A). These results indicate that hair follicles can repair certain degrees of tissue damage on their own. As culture time increased, the number of newly generated cells across all injured tissues increased, and the proportion of newly generated cells also increased significantly (*p* < 0.05), suggesting that cell proliferation plays a critical role in the hair follicle repair process. Comparing the proportion of proliferating cells in different injured tissues of hair follicles at various culture times revealed that the outer root sheath exhibited the fastest increase in cell proliferation rate, with a significantly higher proliferation rate than other injured tissues at 3 days of culture. These experimental results demonstrate that outer root sheath cells exhibit robust proliferative capacity, as shown in Figure 4B–H. The outer root sheath of freshly dissected hair follicles consisted of a layer of tightly arranged keratinocytes, with no cells remaining in the center. After culture, however, the center of the outer root sheath showed a large number of cells and extracellular matrix, with a reduced blank area. These cells were approximately oval-shaped, with some undergoing different stages of mitosis, and the outer layer of cells began to form an encircling pattern. These results further confirm the importance of cell proliferation in hair follicle regeneration and repair (Figure 4Q,R).

With prolonged culture time, the positive signals for CD34 and SOX9 across all injured tissues gradually intensified, and the average fluorescence intensity increased significantly (*p* < 0.05). During the hair follicle repair process, the expression levels of CD34 and SOX9 proteins increased, the number of hair follicle stem cells rose, and their proliferative and differentiation capabilities were enhanced. This indicates that hair follicle stem cells can promote the repair of injured hair follicles. Furthermore, comparing the average fluorescence intensity of CD34 and SOX9 in different injured tissues of hair follicles revealed that the expression levels of CD34 and SOX9 proteins in the outer root sheath were significantly higher than those in other injured tissues (*p* < 0.05). This suggests that hair follicle stem cells in the outer root sheath exhibit stronger proliferative and differentiation abilities. Therefore, subsequent experiments will select the outer root sheath as the research material to investigate the impact of the Wnt/β-catenin signaling pathway on hair follicle stem cell function, as illustrated in Figure 4I–P.

### 3.3. Activation of the Wnt/β-Catenin Signaling Pathway Enhances the Proliferative Capacity of Outer Root Sheath Cells

**Figure 5:**
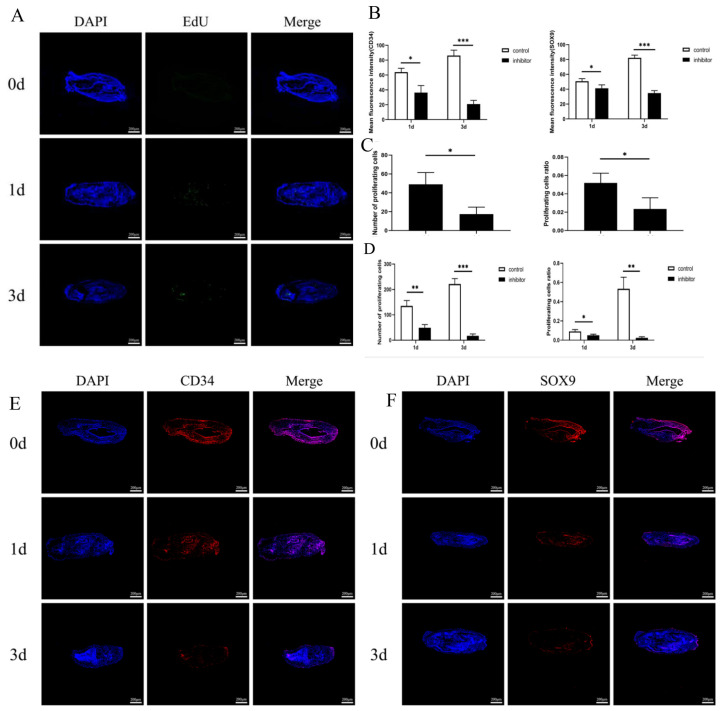
(**A**) Detection of proliferating cells in the outer root sheath of hair follicle cultured in vitro of the Wnt/β-catenin signaling pathway inhibition group. Green: EdU; Blue: Nucleus (DAPI); Scale: 200 μm. (**B**) Comparison of CD34 and SOX9 mean fluorescence intensity (MFI) between the control and inhibitor groups. Left: MFI statistics for CD34; Right: MFI statistics for SOX9. *: *p* < 0.05; ***: *p* < 0.001. (**C**,**D**) Changes in the number and proportion of proliferating cells in the outer root sheath inhibitor group, as well as a comparison of differences with the control group. (**C**) Changes in the number and proportion of proliferating cells within the inhibitor group. *: *p* < 0.05. (**D**) Comparison of the number and proportion of proliferating cells between the control group and the inhibitor group. *: *p* < 0.05; **: *p* < 0.01; ***: *p* < 0.001. (**E**,**F**) Immunofluorescence staining of CD34 and SOX9 in the outer root sheath of the Wnt/β-catenin signaling pathway inhibition group. Red: CD34, SOX9; Blue: Nucleus (DAPI); Scale: 200 μm.

After the addition of the inhibitor, the wound in the outer root sheath tissue caused by the removal of the inner root sheath remained present on day 1 of culture, and the incision at the base of the outer root sheath had not yet closed (Figure 4S–U). By day 3 of culture with the inhibitor, although the incision in the outer root sheath had closed, a significant amount of tissue debris remained in the center. Compared with normal outer root sheath culture results, inhibition of the Wnt/β-catenin signaling pathway delayed hair follicle repair, indicating that this pathway regulates hair follicle repair. Due to the inhibition of the Wnt/β-catenin signaling pathway, the number and proportion of proliferating cells in the outer root sheath of the inhibitor group were significantly lower than those in the control group (*p* < 0.05, Figure 5D). Additionally, the number and proportion of proliferating cells within the inhibitor group were also significantly reduced. These findings suggest that the Wnt/β-catenin signaling pathway regulates cell proliferative capacity, and its inhibition weakens this capacity, reduces the cell proliferation rate, and consequently slows the wound-healing process in the outer root sheath. The results are shown in Figure 5A,C.

In the inhibitor group, the intensities of CD34- and SOX9-positive signals decreased with prolonged culture time, and the areas of positive signals gradually decreased (Figure 5E,F). The average fluorescence intensities of CD34 and SOX9 in the inhibitor group were significantly lower than those in the control group (*p* < 0.05, Figure 5B). Furthermore, the average fluorescence intensity of CD34 and SOX9 in the inhibitor group showed a clear decreasing trend. This indicates that the expression levels of the CD34 and SOX9 proteins in the inhibitor group were lower than in the control group and continued to decline over time. These results demonstrate that the Wnt/β-catenin signaling pathway participates in the hair follicle damage repair process by regulating the activity of hair follicle stem cells. Activation of the Wnt/β-catenin signaling pathway can mediate hair follicle stem cells to promote the repair of damaged hair follicles.

**Figure 6:**
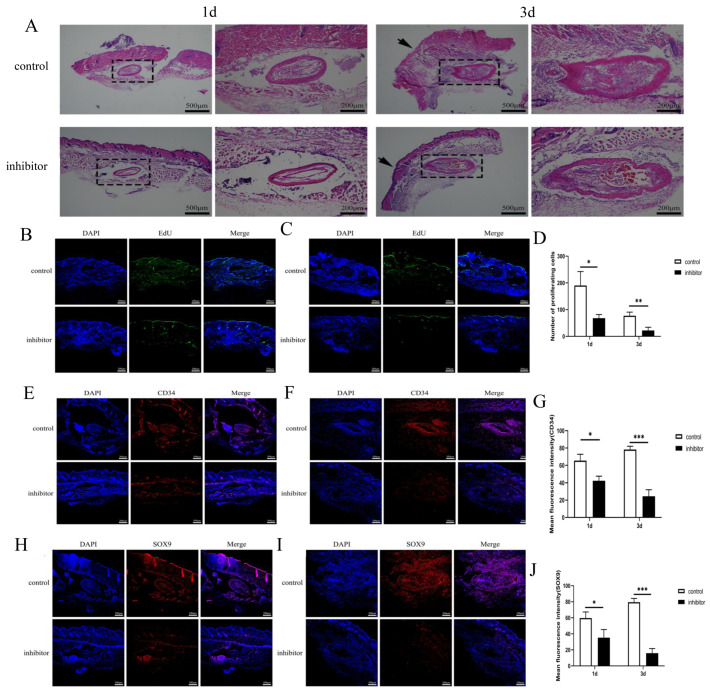
(**A**) Changes in tissue morphology of the outer root sheath transplantation area under different treatments. Dashed box: Location of partial magnification; Black arrow: Location of skin wound; Complete transplantation area scale: 500 μm; Partial magnification scale: 200 μm. (**B**–**D**) Comparison of the number of proliferating cells and migration changes in the outer root sheath between the control group and the inhibitor group. (**B**) Skin tissue after outer root sheath transplantation for 1 day; (**C**) Skin tissue after outer root sheath transplantation for 3 days. Green: EdU; Blue: Nucleus (DAPI); Scale: 200 μm. (**D**) Statistics on the number of proliferating cells. *: *p* < 0.05; **: *p* < 0.01. (**E**–**G**) Comparison of CD34 mean fluorescence intensity in the transplant area between the control group and the inhibitor group. (**E**) Skin tissue after outer root sheath transplantation for 1 day; (**F**) Skin tissue after outer root sheath transplantation for 3 days. Red: CD34; Blue: Nucleus (DAPI); Scale: 200 μm. (**G**) Statistical analysis of CD34 mean fluorescence intensity (MFI), *: *p* < 0.05; ***: *p* < 0.001. (**H**–**J**) Comparison of SOX9 mean fluorescence intensity in the transplant area between the control group and the inhibitor group. ((**H**) Skin tissue after outer root sheath transplantation for 1 day; (**I**) Skin tissue after outer root sheath transplantation for 3 days. Red: SOX9; Blue: Nucleus (DAPI); Scale: 200 μm. (**J**) Mean fluorescence intensity statistics of SOX9. *: *p* < 0.05; ***: *p* < 0.001.

One day after outer root sheath transplantation, the incisions in the outer root sheath of both the control and inhibitor groups had closed. Compared with the inhibitor group, the outer root sheath in the control group began to form connections with surrounding tissues. As the transplantation time increased, the connection between the outer root sheath and the surrounding skin tissues deepened in both groups, and the skin wounds caused by transplantation had also healed (Figure 6A). However, the tissue formed at the wound site in the control group was more complete, whereas in the inhibitor group, only the epidermal layer of the skin wound had healed, and the healed area was relatively fragile. By day 3 after transplantation, the wound in the outer root sheath of the control group, caused by removal of the inner root sheath, had closed and filled with cells. In contrast, the outer root sheath of the inhibitor group still contained substantial cell debris and tissue gaps. These results indicate that the outer root sheath can serve as a graft to repair damaged skin, and that inhibition of the Wnt/β-catenin signaling pathway weakens its repair efficacy. This demonstrates that activation of the Wnt/β-catenin signaling pathway is crucial for outer root sheath-mediated skin repair.

As the skin wounds healed, the number of proliferating cells in the outer root sheath of both groups decreased, with proliferating cells primarily observed in the epidermal layer (Figure 6B–D). Compared with the control group, the inhibitor group exhibited weaker positive signals and a significant reduction in the number of proliferating cells at 1 and 3 days post-transplantation (*p* < 0.05). Hair follicle outer root sheath cells were able to migrate to the epidermal layer of the skin to promote wound healing. CD34 and SOX9 positive markers were widely distributed in the transplantation areas of both groups. The control group showed increased CD34- and SOX9-positive signals, whereas the inhibitor group showed reduced CD34- and SOX9-positive signals. Compared with the control group, the average fluorescence intensities of CD34 and SOX9 in the inhibitor group at 1 and 3 days post-transplantation were significantly lower (*p* < 0.05). Meanwhile, as the skin wounds healed, the expression levels of CD34 and SOX9 proteins in the control group showed an increasing trend (Figure 6E–J). These findings indicate that activation of the Wnt/β-catenin signaling pathway can enhance the proliferative capacity of outer root sheath cells and accelerate skin injury repair.

## 4. Discussion

### 4.1. Discussions About the Study

As a miniature organ on the skin, the hair follicle has a complex, delicate structure and performs multiple important physiological functions. The renewal of stem cells within the hair follicle drives the hair follicle cycle and maintains the homeostasis of ex vivo hair follicle tissues. Moreover, hair follicle stem cells are often used as seed cells in tissue engineering. Our study demonstrates that the bulge, inner root sheath, and dermal papilla of hair follicles grow with hair follicle development, with these structures exhibiting faster growth rates on day 7. During hair follicle growth, the number of newly generated cells decreases, while the functionality of hair follicle stem cells enhances. Hair follicle stem cells achieve their biological functions through the regulation of the Wnt/β-catenin signaling pathway. The Teplinovivint inhibitor significantly suppresses Wnt/β-catenin signaling, thereby impairing the capabilities of hair follicle stem cells. However, this study only intervened with pharmacological inhibitors and failed to verify the pathway’s necessity using genetic manipulation (such as conditional gene knockout or mutation). Inhibitors may have off-target effects and cannot achieve cell-type-specific regulation. Future research should combine CRISPR/Cas9-mediated gene editing to achieve knockout or knock-in of target genes in specific cell populations and conduct functional rescue experiments to establish the rigor of causal relationships.

The CD34 molecule is a highly glycosylated cell-surface antigen initially identified on hematopoietic stem cells and endothelial cells and has since been widely detected in hair follicles [19,20]. CD34 is widely recognized as a stem cell surface marker, expressed on various stem cell types and playing a critical role in their proliferation and differentiation. This makes CD34 an important research tool for identifying and isolating stem cells in fields such as tissue engineering, regenerative medicine, and stem cell therapy. Hair follicles contain CD34-expressing stem cells that contribute to new hair growth and regeneration, playing a vital role in maintaining hair health and the hair growth cycle. The SOX9 protein is a key transcription factor and a critical component in the regulation of reproductive system development. It plays essential roles in cell differentiation, embryonic development, organ formation, and tissue regeneration. SOX9 is pivotal in regulating the proliferation and differentiation of hair follicle stem cells [21]. It helps maintain stem cell characteristics and promotes their differentiation into various hair follicle cell types [22]. SOX9 is also involved in regulating multiple signaling pathways during hair follicle development, playing a significant role in hair follicle formation, growth, and cyclic regeneration. SOX9 is of great importance in hair follicle biology research. This study found that the expression of both CD34 and SOX9 increases with hair follicle growth, suggesting a potential association between these stem cell markers and hair follicle development. While these findings are consistent with a promotive role of hair follicle stem cells in hair growth, they do not directly demonstrate enhanced proliferative or differentiation capacity. Therefore, our conclusions regarding differentiation remain preliminary. Based on current research findings, in addition to hair follicle stem cells, the outer root sheath protrusions also contain other types of stem cells, including melanin stem cells, neural crest stem cells, and epidermal stem cells. Among them, melanin stem cells can proliferate and differentiate into mature melanocytes, possessing the functions of pigment regeneration and maintenance of tissue homeostasis. Neural crest stem cells are a type of pluripotent stem cell that can differentiate into various cell types. Epidermal stem cells are involved in re-epithelialization and participate in skin damage repair under the regulation of the nervous system. Epidermal stem cells exist in two parts of the hair follicle. Epidermal stem cells labeled with Lgr6 are stored in the isthmus of the hair follicle and the sebaceous gland, while those labeled with Gli1 are expressed at the upper end of the hair follicle bulge. This study involved the transplantation of the entire outer root sheath and has not yet performed lineage tracing to determine the type of stem cells involved. Therefore, in subsequent research, the lineage origin will be further explored. For instance, neural crest stem cells isolated from hair follicles will be identified by naspin- and CD34-positivity and CK15-negativity.

Outer root sheath transplantation is a hair follicle transplantation method commonly used to treat hair disorders and is now also being employed in studies investigating skin repair. Transplanting the outer root sheath involves extracting healthy hair follicles, dissecting the remaining outer root sheath, and transplanting it to the damaged skin site. Skin tissue engineering promotes wound healing by transplanting skin analogs containing stem cells. As hair follicles are miniature organs rich in stem cells and capable of maintaining tissue homeostasis ex vivo, they can serve as natural stem cell-based biomaterials for treating skin diseases. This study found that hair follicle stem cells in the outer root sheath enhance their proliferation, differentiation, and migration capabilities by activating the Wnt/β-catenin signaling pathway, thereby promoting skin wound healing. The bulge region of the outer root sheath, acting as a stem cell niche, is the primary storage site for hair follicle stem cells. These stem cells remain quiescent or slowly renewing for most of the time in intact hair follicles. However, damage to the outer root sheath activates hair follicle stem cells, increasing stem cell production and, in turn, facilitating tissue repair. Thus, this study demonstrates the feasibility of using transplanted outer root sheaths to repair skin wounds, providing a new tool for the clinical treatment of skin injury-related diseases. Of course, since the samples come from 7-day-old newborn mice, the differentiation stage of the nascent vibrissae follicles may differ from the cycle stage of adult hair follicles. Neonatal tissues have greater regenerative potential and lower immunogenicity, which limits the generalizability of the results to adult or pathological environments. Future research should obtain outer root sheaths from adult donors in adult mice, aged mice or chronic wound models (such as diabetic mice) for transplantation to evaluate their efficacy in different contexts. The observation period of this study was insufficient to assess the graft’s long-term stability, functional maintenance, or long-term adverse events (such as excessive scar hyperplasia or malignant transformation). Future studies should design long-term follow-ups of at least 12 weeks and conduct comprehensive evaluations using histological, immunohistochemical, and functional indicators (such as skin tensile strength and hair follicle cycle recovery).

### 4.2. Limitations of the Study

Our data establishes the role of the Wnt/β-catenin signaling pathway in mediating the effects of outer root sheath stem cells on wound healing. Firstly, we verified only one signaling pathway, did not explore cross-dialogue or alternative pathways, and most mechanistic conclusions relied on the expression patterns of endpoint markers. Future research should integrate phosphorylated proteomics, transcriptome sequencing (scRNA-seq), and a multipathway inhibitor/activator matrix to comprehensively analyze the molecular network by which the outer root sheath regulates wound healing, and conduct bidirectional verification through function acquisition and function loss experiments. Secondly, we mainly evaluated the wound closure rate and the expression of some markers. Still, we lacked the detection of key indicators for functional healing, such as the rate of re-epithelialization, the maturity of neovascularization, the quality of collagen remodeling, the regeneration of skin appendages (hair follicles, glands), and mechanical property tests. Future research should incorporate a multi-point, quantitative assessment of healing quality, including transdermal water loss, dermal collagen alignment (e.g., Sirius red staining under polarized light), and nerve re-innervation. Finally, a limitation of this study is the lack of blinding during image analysis, which may have introduced observer bias. This was unavoidable, however, because the morphological differences between groups were still easily discernible after anonymization. To mitigate this bias, all measurements were independently made by two researchers, with excellent inter-rater reliability (ICC > 0.90).

## 5. Conclusions

Hair follicle stem cells promote the growth of skin and hair follicles through their abilities to proliferate, differentiate, and migrate. Damage to the skin and hair follicles can activate hair follicle stem cells, thereby facilitating the repair of injured tissues. Activation of the Wnt/β-catenin signaling pathway can promote hair follicle stem cell proliferation and differentiation, thereby promoting hair follicle growth, repair of damaged hair follicles, and skin wound healing. Meanwhile, studies have found that the outer root sheath of hair follicles, as a graft with inherent tissue-engineering properties, can be applied to future clinical research on the treatment of related skin diseases. Of course, our findings, while derived from short-term neonatal mouse experiments, provide preliminary evidence that outer root sheath transplantation merits further investigation. Whether this approach could ever be relevant to clinical hair restoration remains unknown and would require extensive long-term studies in adult animal models and, ultimately, human trials.

## Figures and Tables

**Table 1:** Statistics on the area of each part of the hair follicle at different growth times (μm^2^).

Part of the Hair Follicle	Time		
	3 d	7 d	15 d
Hair follicle bulge	4420.65 ± 1485.47 c	8173.9 ± 1038.01 b	11,935.07 ± 1384.87 a
Inner root sheath of hair follicle	18,587.36 ± 2123.39 c	28,946.07 ± 5252.48 b	50,314.89 ± 2121.84 a
Dermal papilla of hair follicle	3645.7 ± 788.41 c	5828.76 ± 803.66 b	10,867.84 ± 2014.94 a

Peer data with the same letter indicate no significant difference (*p* > 0.05), while different lowercase letters indicate a significant difference (*p* ≤ 0.05).

## Data Availability

The original contributions presented in this study are included in the article/Supplementary Materials. Further inquiries can be directed to the corresponding authors.

## Supplementary Materials

The following supporting information can be downloaded at: https://www.mdpi.com/article/10.3390/cells15111038/s1. Figure S1. Reversal of Wnt/β-catenin pathway activity by Wnt activators. (A) Western blot analysis of different treatment groups. Expression levels of β-catenin and Wnt3a in the Wnt inhibitor group and the inhibitor + activator group on day 1, day 3, and day 5; GAPDH was used as an internal control. (B–C) Grayscale quantitative analysis of the relative protein levels. (Data are expressed as Mean ± SD. * *p* < 0.001. The Wnt inhibitor group at the same time point. Figure S2. Regulatory effect of the Wnt activator on AXIN2, a downstream target gene of the Wnt/β-catenin pathway. (A) Western blot analysis of different treatment groups. Expression levels of AXIN2 protein in the Wnt inhibitor group and the inhibitor + activator group on day 1, day 3, and day 5; GAPDH was used as an internal control. (B–D) Grayscale quantitative analysis results. Data are expressed as Mean ± SD. ***: *p* < 0.001. The Wnt inhibitor group at the same time point.
