# Supplementary material for: Role of the Wnt/β-Catenin Signaling Pathway in Mediating Outer Root Sheath Stem Cells to Promote Hair Follicle Regeneration and Skin Wound Healing in Mice
Source: Cells. 2026 Jun 5;15(11):1038. doi: 10.3390/cells15111038 (PMC13257427; doi:10.3390/cells15111038)
Supplement: Supplementary file 1 [file cells-15-01038-s001.zip › cells-4302304-supplementary.pdf]

Adding a Wnt inhibitor during hair follicle culture effectively suppressed the expression of hair follicle-associated proteins. Subsequently, a rescue experiment was performed by washing out the inhibitor and then adding a Wnt activator to continue culturing the hair follicles. This approach effectively reversed the pathway inhibition, ruled out non-specific interference, and confirmed that the Wnt/β-catenin pathway has a direct causal regulatory relationship with the target phenotype.

The regulatory effect of the Wnt activator on key proteins of the Wnt/β-catenin pathway. Following continuous treatment with the Wnt inhibitor, addition of the Wnt activator significantly upregulated the protein expression levels of Wnt3a and β-catenin, as detected on days 1, day 3, and day 5. This upregulation showed an increasing trend with longer treatment time (Figure S1A–C). Grayscale quantitative analysis revealed that, compared with the inhibitor group at the same time point, the differences in Wnt3a and β-catenin expression in the activator-treated group were extremely statistically significant ($p < 0.001$). These results indicate that the Wnt activator can effectively reverse the inhibitory effect of the inhibitor on the Wnt pathway and successfully activate the canonical Wnt/β-catenin signaling pathway.

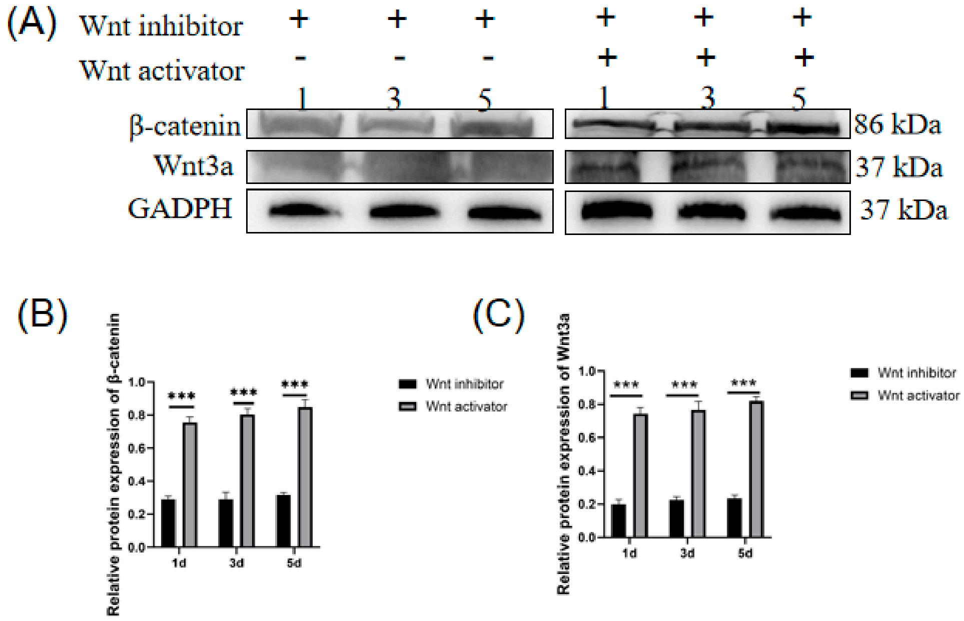

Figure S1. Reversal of Wnt/β-catenin pathway activity by Wnt activators. (A) Western blot analysis of different treatment groups. Expression levels of β-catenin and Wnt3a in the Wnt inhibitor group and the inhibitor + activator group on day 1, day 3, and day 5; GAPDH was used as an internal control. (B–C) Grayscale quantitative analysis of the relative protein levels. (Data are expressed as Mean ± SD. *$p < 0.001$. The Wnt inhibitor group at the same time point.

We performed western blot detection of AXIN2, a direct downstream target gene of the Wnt pathway. The results show that adding a Wnt activator following inhibitor treatment significantly upregulates AXIN2 protein expression in a time-dependent manner. These findings directly confirm activation of the canonical Wnt/β-catenin pathway at the transcriptional level, overcoming the limitation of detecting only upstream proteins and providing more rigorous evidence for pathway activation.

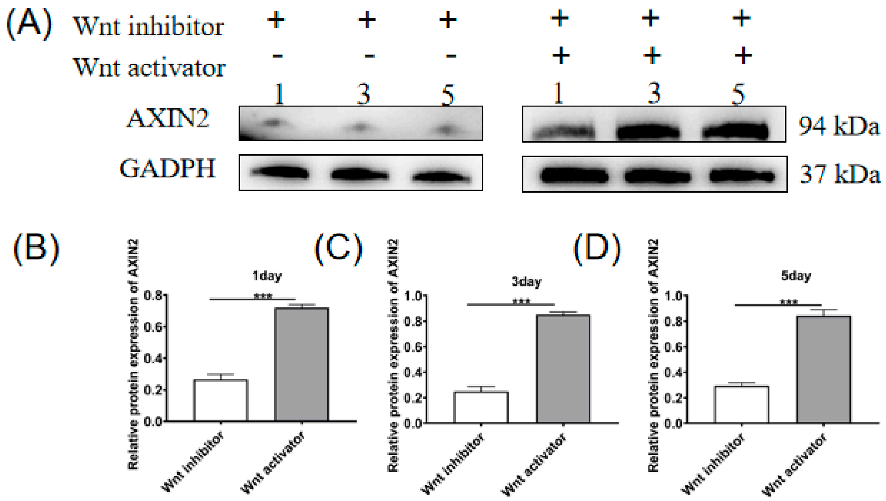

Figure S2. Regulatory effect of the Wnt activator on AXIN2, a downstream target gene of the Wnt/β-catenin pathway. (A) Western blot analysis of different treatment groups. Expression levels of AXIN2 protein in the Wnt inhibitor group and the inhibitor + activator group on day 1, day 3, and day 5; GAPDH was used as an internal control. **(B–D)** Grayscale quantitative analysis results. Data are expressed as Mean ± SD. ***: $p < 0.001$. The Wnt inhibitor group at the same time point.

The regulatory effect of Wnt activators on AXIN2, a direct downstream target gene of the canonical Wnt/β-catenin pathway. Following continuous treatment with a Wnt inhibitor, addition of a Wnt activator significantly upregulated AXIN2 protein expression at day 1, day 3, and day 5, and this upregulation increased with treatment duration (Figure S2A). Grayscale quantitative analysis revealed that, compared with the inhibitor group at the same time points, the differences in AXIN2 expression in the activator-treated group were highly statistically significant ($p < 0.001$; Figures S2B–D). These results directly confirm at the transcriptional level that Wnt activators can effectively reverse the inhibitory effect of the inhibitor on the Wnt pathway and successfully activate the canonical Wnt/β-catenin signaling pathway.
